# Periodontal Infectogenomics

**DOI:** 10.1186/s41232-018-0065-x

**Published:** 2018-05-07

**Authors:** Gurjeet Kaur, Vishakha Grover, Nandini Bhaskar, Rose Kanwaljeet Kaur, Ashish Jain

**Affiliations:** Department of Periodontology, Dr Harvansh Singh Judge Institute of Dental Sciences and Hospital, Panjab University, Sector-25, Chandigarh, India

**Keywords:** Infectogenomics, Genetics, Microbes, Periodontitis, Bacterial species, Invasion, Proliferation

## Abstract

Periodontal diseases are chronic infectious disease in which the pathogenic bacteria initiate the host immune response leading to the destruction of tooth supporting tissue and eventually result in the tooth loss. It has multifactorial etiological factors including local, systemic, environmental and genetic factors. The effect of genetic factors on periodontal disease is already under extensive research and has explained the role of polymorphisms of immune mediators affecting disease response. The role genetic factors in pathogens colonisation is emerged as a new field of research as “infectogenomics”. It is a rapidly evolving and high-priority research area now days. It further elaborates the role of genetic factors in disease pathogenesis and help in the treatment, control and early prevention of infection. The aim of this review is to summarise the contemporary evidence available in the field of periodontal infectogenomics to draw some valuable conclusions to further elaborate its role in disease pathogenesis and its application in the clinical practice. This will open up opportunity for more extensive research in this field.

## Background

Periodontal disease is a highly prevalent, multifactorial, chronic inflammatory disease of periodontium eventually leading to destruction of supportive tissues of teeth and tooth loss. The interaction between microbes present in dental plaque and host immune response is a major determinant of progression and clinical manifestations of periodontal disease [1, 2]. However, there are multitude of factors like systemic, environmental and genetic which directly or indirectly influence this association at multiple levels [3, 4]. It has been seen that individuals harbouring almost equivalent local etiological factors could represent the diverse disease severity. These observations lead to the idea of some unrecognised components of the host genetic constitution or the environment which was responsible for differences in their susceptibility of disease [5, 6]. The disease susceptibility is determined by immune response of the body as applies to periodontal disease, which is largely determined by genetic or epigenetic factors [6, 7]. The effects of these factors have been extensively studied over the last 20 years using different study designs. This has resulted in a significant paradigm shift in the aetiology of periodontal disease with the increased emphasis on host and its genetic constitution as modifiers of the bacterially induced disease and for increased risk of disease occurrence and severity.

A huge published literature is available regarding genetic analysis using candidate gene and human leukocyte antigen (HLA) markers for periodontitis among which the polymorphism studies of genes coding for cytokines have received the most attention [7]. Lot of investigations have been conducted to identify specific gene polymorphisms associated with risk for periodontal diseases. No specific single gene polymorphism could be defined, owing to polygenic nature of disease [6].

The altered immune response due to these gene polymorphisms affects the microbial composition present in periodontal environment. Humans are considered supra-organisms consisting of trillions of symbiotic, commensal and pathogenic bacteria [8, 9]. The oral cavity contains over 1000 different microorganisms including 700 different species with as many as 19,000 different bacterial phylotypes which are mostly commensal in nature [3, 8]. A myriad of host factors is responsible for the development of composition of oral microbiome, its role in oral health and disease which further shows subject to subject variation [8, 10]. Many polymorphisms affecting the immune response have been linked to periodontal disease which indirectly may have an impact on the quantity and quality of microbial colonisation [3]. This has led to a novel concept in aetiology of periodontal disease i.e. “infectogenomics”.

## The concept of infectogenomics

*Infectogenomics was first defined by Kellam and Weiss (2006) as the study of interaction between host genetic variations and colonisation by pathogenic microbes* [11]. This term is in line with the word pharmacogenetics in which the appearance of a disease or symptom following exposure to an infectious agent can be regarded as an unusual side effect just like an adverse reaction to a drug [11]. With the alteration in the host genotype, these adverse reactions can be severe in one person as compared to the other.

The concept for infectogenomics states that the genetic defects in the recognition and response pathways of the host to identify microbial pathogens predispose to either altered microbial colonisation or misrecognition of normal microbiota leading to dysbiosis and appearance of infectious disease [9]. This hypothesis of association between host genomic adaptations and microbiome is well studied in many systemic disease conditions. A specific disease endemic to a particular population is known to cause certain genetic mutations as a result of this selective disease susceptibility and renders the population in subsequent generations resistant in due course of time. The classical example of this concept of selective pressure is studied in malaria endemic areas where the modifications in the human haemoglobin genes make this population resistant to malaria [12, 13]. Such mechanisms strongly support the concept of genetics linking disease susceptibility. Converse to this the concept of infectogenomics which suggests the reverse relationship that certain genetic constitutions are particularly susceptible to the disease and this is mediated by the selective pressure in terms of microbial colonisation or proliferation [3, 14, 15].

The genomic adaptations of the host can have effect either on the pathogen invasion or on pathogen proliferation [3, 9]. After the invasion of pathogens in the human body, the interaction between the pattern recognition receptors (PRRs) and the pathogen associated molecular patterns (PAMPs) generate cellular signalling against microbes. Any mutation or modifications in the PRR genes may thus result in either its altered expression or affect its ability to recognise microbial constituents effecting invasion of pathogens in the host [9]. A well documented association is between the CCR5-∆32 deletion allele and human immunodeficiency virus (HIV) resistance. CCR5 chemokine receptor is used by HIV strains to gain entry into immune system cells. So, CCR5-∆32 deletion allele provided almost complete resistance against HIV-1 in homozygous state and partial resistance with slower disease progression in heterozygous state [16–18]. It was hypothesised that this modification in the genetic constitution arose in high risk population due to selective pressure from bubonic plague or small pox [17]. Next step in the pathogenesis is the proliferation of the pathogens that trigger immune-pathological reactions which determines the severity and progression of infectious disease. Inflammation being a central mechanism in many chronic human diseases, any alteration in immune regulatory genes may affect the disease pathogenesis. Selective genetic variations may result in skewing the microbial composition toward more pathological microbes or alters the host response for developing resistance for a particular pathogen [9]. This mechanism is well documented as conferring resistance to malaria in subjects with haemoglobin S (HbS) variant in malaria endemic population [12, 13]. In the individuals presenting with HbS homozygous traits the presence of *Plasmodium falciparum* causes the red cells to rupture, thus inhibits its proliferation or colonisation [19]. Converse to this is a well studied association between the cystic fibrosis and *Pseudomonas aeruginosa* infection in which the ∆F508 mutation in cystic fibrosis transmembrane conductance regulator (CFTR) gene lead to hypersusceptibility to chronic lung infection due to alterations in pH, ion concentrations and formation of dehydrated airway surface layer which contributes to increased proliferation of *Pseudomonas aeruginosa* [20, 21]. Other examples are protective role of T-helper cells type 2 (Th2) immune responses against Schistosomiasis [12], polygenic susceptibility to tuberculosis [22] and protection against chronic viral hepatitis [23]. However, in case of extensively studied inflammatory bowel disease the genetic mutations have affect on both the pathogen invasion and proliferation; explains the bi-directional relationship of the microbiome interactions with host genetics as between altered host immune function and altered bacterial community functions, features or by-products [15, 24]. So, these medical evidences provide us with some clear patterns of associations emerging in the field of infectogenomics.

## Periodontal infectogenomics

With the advancement in the research it has been seen that the most prevalent chronic periodontitis being multifactorial also entails a dysbiotic oral microbial shift and a deregulated host inflammatory response resulting in progressive periodontal tissue destruction [10, 25]. Earlier the main focus for the genetic analyses was the association of periodontal disease with altered immune response taking into consideration some candidate genes related to immune pathways. In the past 15 years a lot of research work is mainly focused on this new concept of periodontal infectogenomics which will potentially help to better understand the pathogenesis of periodontal disease.

### Genetic factors affecting periodontal pathogen invasion

In periodontal environment, microbes causing infection must have the ability to attach to the tissue surface, to multiply, to compete against other microbial species and to defend against host responses [3, 9]. One of the key systems for immune surveillance is complement system which links the innate and the adaptive arms of the host immune response [26, 27]. In monogenic Ehler Danlos Syndrome the alterations in C1R or C1S genes encoding for complement 1 subunits C1r and C1s has been documented as a link between connective tissue pathology with classical complement pathway [28]. Integrative gene prioritisation method has listed C3 among the top 21 most promising candidate genes involved in periodontitis (Polygenic condition) [26, 29]. Animal model investigations have indicated that complement is involved in both the dysbiotic transformation and the inflammatory response that leads to destruction of periodontal tissue. Similar findings have been reported in human clinical and histological studies [26]. In Hong Kong Chinese population single nucleotide polymorphism of C5 (rs17611) with genotype AG and the haplotype CGCA containing rs1035029, rs17611, rs25681 and rs992670 has been found to be significantly more prevalent in periodontitis patients than in healthy controls [27, 30]. Only the MBL2 homozygote (O/O) variant type, a secreted pattern-recognition molecule in the cascade of lectin pathway, could provoke the virulence of *A. actinomycetemcomitans* with no difference found between *P. gingivalis* and/or inflammatory markers in saliva and periodontal tissue destruction in study subject [31].

The mutations in few pattern recognition receptors including toll like receptors (TLRs), NOD-like receptors (NLRs), formyl peptide recptors and Fc receptors have been studied so far to express the alteration or the misrecognition of microbial constituents resulting in altered response to microbes. The effect of genetic polymorphisms in these receptors is mainly expressed as their response characteristics at the protein and mRNA level after exposure to various cytokines and microbes. The *CD14* -260CT + TT genotype is found to have higher frequencies of red complex bacteria i.e. *Porphyromonas gingivalis, Treponema denticola* and *Tannerella forsythia* particularly in renal transplant patients with cyclosporine A induced gingival overgrowth which is associated with high interleukin-1β (IL-1β) levels [32]. The altered immune response due to immunosuppressive medication and disruption of normal symbiotic relation can be plausible explanation for these findings. In contrast, the CD14 -159TT variant has found to have a protective role in periodontitis patients by reducing subgingival colonisation of *Prevotella intermedia* [33]. In the healthy individuals the mutant type of *TLR 4* (Asp299Gly heterozygote) has been appeared less responsive to *Porphyromonas gingivalis* than wild type TLR4(normal) [34]. But no association has been seen with TLR4 polymorphisms (Asp299Gly and Thr399Ile) in periodontitis patients in relation to subgingival occurrence of periodontopathogens [33]. Similarly, TLR 2 polymorphism (− 16,934 T/A) in both healthy and periodontitis patients has shown no association [35]. In Czech population, the TLR-9 haplotype -1486 T/− 1237 T/+2848A has been found to increase the susceptibility of chronic periodontitis but without affecting the subgingival colonisation of bacteria [35]. Another, extensively studied receptor is *Fc receptors* only in two alleles i.e. *FcγRII* and *FcγRIII* types. FcγRIIa131H/H genotype has been found to be hyper-reactive phenotype of the polymorphonuclear neutrophils (PMNs), which release more bioactive molecules in response to periodontal pathogens and aggravate the periodontal destruction [36, 37]. In contrary to this the FcγRIIb-nt645 + 25AA genotype has been seen to be linked with more severe periodontitis in Japanese population, due to suppression of humoral response against periodontopathic [38, 39]. Similarly, inefficient phagocytosis of bacteria by neutrophils in FcγRIIIb-NA2 subjects is responsible for an increased levels of bacteria in gingival crevice leading to high risk of periodontitis [40]. Most of the investigations were documented in chronic periodontitis, only a single polymorphism studied in relation to aggressive periodontitis is nt324 A/A FcαRI polymorphism. It exhibited similarly a decreased phagocytosis of periodontopathic bacteria *Porphyromonas gingivalis* in Japanese population [41]. This body of literature revealed that functional differences in the activity of immune cells possibly lead to inter individual differences in the subgingival colonisation of periodontal pathogens and the development of periodontitis.

### Genetic factors affecting periodontal pathogen proliferation/ clearance

The recognition of invaded periodontopathogens leads to the activation of immune regulatory mechanisms which is deterministic for the onset and progression of periodontal disease. It was hypothesised that alteration in the genetic constitution of components of immune regulatory mechanism can alter the subgingival environment for the proliferation of microbes in both healthy and diseased state of periodontium.

#### Chronic periodontitis

The polymorphisms in the cluster of *IL-1* gene have been the most extensively studied polymorphism as to explore the link of periodontal disease pathogenesis. Infact, a genetic susceptibility kit based on IL-1β polymorphisms has been commercialised. But apart from the direct effect on host defence mechanisms, indirect bearing of the polymorphisms on periodontal microbes also has been documented. The subjects with IL-1A(+ 4845) and IL-1B(+ 3954) genotype have exhibited higher mean counts of subgingival species belonging to red and orange complexes like *Tannerella forsythia, Treponema denticola, Fusobacterium nucleatum* subspecies, *Fusobacterium periodonticum, Campylobacter gracilis, Campylobacter showae, Streptococcus constellatu, Streptococcus intermedius, Streptococcus gordonii* and 3 *Capnocytophaga* species in sites with increasing pocket depth [42]. In contrast, the Caucasian subjects with this single nucleotide polymorphisms (SNPs) presenting periodontitis has demonstrated negative association with the subgingival colonisation of microbes [43–46]. But individually IL-1β + 3954 genotype had exhibited higher prevalence of *Porphyromonas gingivalis*, *Tannerella forsythia* and *Treponema denticola* species in subgingival sites and higher expression of IL-1β mRNA [47]. So, additively both can affect the potential outcome of periodontal disease. In a group of subjects with periodontitis with IL-1A-889 and IL-1B + 3953 genotype the total count of red complex (*Porphyromonas gingivalis*, *Tannerella forsythia* and *Treponema denticola*), orange complex (*Fusobacterium nucleatum*, *Peptostreptococcus micros*, *Prevotella intermedia*, *Campylobacter rectus*) bacteria and of *Campylobacter rectus* has been found to be 3-fold and 2-fold higher than the negative genotype subjects [48]. So, IL-1 genotype is considered as a non-mandatory but a contributable risk factor for periodontal disease progression and no definitive conclusions could be drawn on the effect of this genotype on the individual subject’s overall mean bacterial load or of their colonisation by specific bacterial clusters. However, allelic forms of same gene polymorphism differentially affect the colonisation of same pathogens. As Caucasian individuals with *IL-2* -330,166 TT:TT genotype has presented with a positive association for the subgingival presence of *Porphyromonas gingivalis* and bacteria of the red complex, but individually subjects with interleukin-2166 TT genotype have been more oftenly infected with *Porphyromonas gingivalis* and bacteria of the red complex whereas a decreased occurrence of *Porphyromonas gingivalis* and bacteria of the red complex found in interleukin-2 -330 TG-positive subjects with a decrease in the odds ratio for chronic periodontitis (odds ratio = 0.394) whereas IL2 -166TT and haplotype IL-2 -330,166 TT:TT associated with an increase in odds ratio (odds ratio = 2.82 or 2.97) [49]. Such kind of observations can pave pathway for the use of gene polymorphisms and their haplotype combination as a putative prognostic factors for chronic periodontitis. The level of periodontopathogens *Porphyromonas gingivalis*, *Tannerella forsythia* and *Treponema denticola* has been found to be higher in Caucasians with chronic periodontitis carrying *IL-4* haplotype with *Treponema denticola* detected in higher counts at diseased sites [50]. This was attributed that the gene polymorphisms alter the immune response against pathogens either by promoting the proinflammatory cytokine production or by suppression of anti-inflammatory function. So, the alteration in a single gene can influence the various cytokines by altering dominated arm of immune response as seen for the levels of IL-4 and IL-13 which are influenced by IL-4 receptor complex specially the *IL-4RA Q551R* and associated with diseases such as the Hyper-IgE syndrome, Atopic dermatitis, Asthma, Systemic lupus erythematosus (SLE), Sjörgren syndrom, Systemic scleroderma, and Cutaneous mastocytosis, where an allergic or autoimmune pathogenesis is assumed [51]. At the same time, this alteration in the immune response have an impact on pathogen colonisation with QR + RR polymorphism of IL-4RA Q551R found to be associated with increased presence of *Tannerella forsythia* in same population [52]. The polymorphism have either enhanced the signal transduction inducing a Th2 dominated response which was ineffective against periodontopathogens or decreased signal transduction with a dominated Th1 type of immune response [52]. So, the altered immune response could cause destructive disease even at lower bacterial loads via influencing response to bacteria rather than their counts. As analysed in the diseased sites of AGT/TTC patients of *IL8* gene polymorphisms − 845 T/C, − 738 T/C, −251A/T, + 396 T/G and + 781C/T, a higher levels of *Porphyromonas gingivalis*, *Tannerella forsythia, Treponema denticola* and red complex have been detected as compared to the patients with ATC/TTC genotype presenting similar clinical parameters suggesting the more destructive inflammatory response even after a lower microbial challenge in patients with ATC/TTC genotype [53]. Similarly, the level of pathogens has been found to be higher in the patients without IL-8 haplotype than with the haplotype patients at the diseased site [54]. In Caucasian patients presenting IL-8 + 781CC genotype with chronic periodontitis the destructive frequency of *Tannerella forsythia* was much less explaining the more destructive immune response [55]. However, in relation to *IL-10,* a multifunctional anti inflammatory cytokine, the subjects positive for ACC, ATA and ACA/ATA have been associated with decreased prevalence of *Prevotella intermedia* as compared to GCC/GCC positive subjects [56]. The genetic constitution was associated with low IL-10 production which is responsible for high local immune response against *Prevotella intermedia* implicated in severe periodontal tissue destruction [56]. Most of polymorphisms analysed in relation to chronic periodontitis have an impact mainly on pathogens belonging to red or orange complexes. However, a strong association has been seen with *Aggregatibacter actinomycetemcomitans* in *IL-6* -174GG genotype subjects considering all subject and tooth related factors [57]. The periodontal pathogen colonisation was found to be unaffected by *IL-12* genotype polymorphisms where as *IFN-γ* 874 AA carriers have been documented for decreased odds ratio for the presence of *Aggregatibacter actinomycetemcomitans* in the oral cavity. Moreover, IFN-γ 874TA predisposed to infection with *Prevotella intermedia* in a group of Caucasian subjects presenting with all disease states [58]. IL-12 and IFN-γ are known to bear a significant application as the maintenance of balance between the Th1 and Th2 type of immune responses. IFN-γ 874 AA genotype carriers primarily activate Th2 cells, as a low producer IFN-γ, only few Th1 cells are also activated, making a more pronounced humoral immune response more effective against *Aggregatibacter actinomycetemcomitans*. Conversely, in subjects who expressed the genotype IFN-γ 874 TA, an intermediate IFN-γ production was associated with an unbalanced Th1/Th2 immune response against *Prevotella intermedia* [58]. There was found to be negatively associated relationship of IFN-γ polymorphisms with the periodontal pathogen colonisation in healthy and chronic periodontitis group in Czech population [59].

Many other cytokines gene polymorphisms like tumour necrosis factor-α (TNF-α), HLA- II, nuclear factor kappa β (NF-κβ), Vitamin D receptor, T bet, MMP8, Apolipoprotein E, peroxisome proliferator activated receptor gamma (PPARγ) involved in periodontal disease pathogenesis are also studied in context with periodontal infectogenomics. Only TNF-α -308GG/−238GG haplotype showed more frequent presence of *Prevotella intermedia* in Caucasians [60]. Similar findings have been reported in coronary heart patients with severe periodontitis in carriers positive for AG + AA genotype and A-allele of TNF-α -308G > A with a risk of 1.4 fold [61]. However, no differences have been found in the frequency or in the load of the periodontopathogens investigated in the different TNFA − 308 genotype groups [62]. The TBX21 -1993 T/C polymorphism of key transcription factor T-bet also found to be involved in the impact of Th1 responses but no association has been documented with load of red complex bacteria [63]. Similarly, no association with the subgingival occurrence of pathogens has been found for Taq1 polymorphisms of vitamin D receptor [64], MMP8 -799C/T and + 17C/G variants [65], polymorphisms of Apolipoprotein E [66] and PPARγPro12Ala polymorphism [67]. To delineate further, more well designed and controlled studies are needed to explore the associations between microbes and host genetic constitution.

#### Aggressive periodontitis

The most extensively studied polymorphism is of *IL-6* gene especially in subjects with aggressive periodontitis. IL-6 -174G genotype has been found to be associated with *Aggregatibacter actinomycetemcomitans* in generalised aggressive periodontitis patients and with both *Aggregatibacter actinomycetemcomitans* and *Porphyromonas gingivalis* in IL-6 -174GG and IL-6 -6106AA polymorphisms [68, 69]. A survey of Indian population on the IL-6 -174 polymorphism presented that in addition to *Aggregatibacter actinomycetemcomitans* another bacteria *Capnocytophaga sputigena* belonging to the green complex found in increased counts in periodontal pockets [70]. The haplotype − 174 G, − 572 C, − 1363 G, − 1480 C, and − 6106 A alleles have been reported to be associated with higher detection of *Aggregatibacter actinomycetemcomitans* whereas haplotype − 174 C, − 572 C, − 1363 T, − 1480 G, and − 6106 A alleles have supposedly protective function toward *Aggregatibacter actinomycetemcomitans* colonisation [69]. So, these findings to some extent confirm the hypothesis that complex interactions between the microbiota and host genome can affect the susceptibility to aggressive periodontitis. Such strong association can be explained as mainly due to faster hyperinflammatory immune response and stimulation of the overgrowth of some particular component of opportunistic organisms making the IL-6 hyperproducer individuals prone to increased risk for periodontal tissue destruction. A rare group of Caucasian population with *IL-1α* rs1800587, *IL-1β* rs1143634 genotype and composite genotype (rs1800587_rs1143634), a significant association of genetic variants and the twofold higher risk of subgingival occurrence of *Aggregatibacter actinomycetemcomitans* have been proved [71]. Further, *IL-8* -251TT genotype subjects also presented with the increased odds ratio for presence of *Aggregatibacter actinomycetemcomitans* in same population [55]. However, longitudinal investigations failed to confirm the role of this host- bacterium interplay in pathogenesis of aggressive periodontitis and its relation to IL-1 composite genotype [72]. In Japanese population, the gene polymorphisms of more frequent 5′ flanking region of *IL12RB2* has been associated with higher serum IgG titres against periodontal bacteria *Aggregatibacter actinomycetemcomitans, Capnocytophaga ochracea, Eikenella corrodens, Fusobacterium nucliatum* [73]. This explained the skewing of immune response toward Th2 responses with higher production of immunoglobulins after infection with periodontal bacteria in carrier group. Major histocompatibility complex-II (*MHC-II)* gene polymorphisms in same population presented with a suggestive hypothesis that the determination of the location of atypical BamHI restriction site in the HLA-DQB1 gene might be useful for determining a tendency toward high susceptibility to localised aggressive periodontitis with *Tannerella Forsythia* infection [74]. The *NF-κβ-*94del/del genotype has also presented with positive association to aggressive periodontitis and with the subgingival occurrence of *Aggregatibacter actinomycetemcomitans* in Caucasians [75].

#### In response to Periodonatal therapy

In Caucasian subjects with IL-1A + 4845/ IL-1B-3954 genotype undergoing supportive periodontal therapy, it has been suggested that a lower bacterial load is required in IL-1 gene positive subjects to develop the same level of periodontitis as in IL-1 gene–negative subjects as analysed from the bacterial load at different sites [76]. However, the periodontal therapy has been found to be equally effective and efficient to reduce the counts of periodontopathogens irrespective of their genetic background [50]. Therefore, the response to periodontal therapy has been found independent of the genetic profile of individual.

#### Other periodontal conditions

In order to study the impact of the genetic constitution on implants, a retrospective study in subjects with IL-1α − 889 and IL-1β + 3953 polymorphisms has been reported to be associated with higher implant loss in synergism with the smoking but without any alteration in microbial colonisation [77].

Among the rare conditions, the IL-10 SNPs has been analysed in renal transplant patients with cyclosporine-A induced gingival overgrowth in a Chinese population and found to be associated with the higher prevalence of *Porphyromonas gingivalis* and *Treponema denticola* especially in subjects with ATA haplotype [78]. So, the low IL-10 expression amplifies the local inflammatory response contributing to development of gingival overgrowth which favours the overgrowth of periodontal pathogens mainly *Porphyromonas gingivalis* and *Treponema denticola*.

A hypothesis has been made to explain the role of infectogenomics in perio- systemic relationship mainly in type 2 diabetes mellitus in association with IL-1 genotype polymorphisms and suggested that dental plaque remains the major contributory factor to progressive periodontitis with periodontal interleukin-1 gene polymorphisms and differences in oral microbiota seem to play only a subordinate role [79].

Through the discrete result from all the studies are difficult to be drawn and the mechanisms yet to explain further. However, the possible biological explanations have been put forth in literature as [42, 45]:

- The cytokines might directly affect the growth and/or virulence activity of bacterial species.
- Indirect mechanism considers that an increased inflammatory response to a given microbial challenge occurs due to an over-production of cytokines. An increased gingival crevice fluid flow in response to inflammation might foster increased levels of subgingival species, particularly species of the red and orange complexes. So, increased levels of these species in turn may affect the local tissues leading to increased inflammation and pocket formation.
- Both the overall lower serum antibody levels and specific titers against selected bacteria have responsible for their colonisation.

### Genome wide association studies in context of periodontal Infectogenomics

The concept of periodontal infectogenomics has been investigated in genome wide association studies (GWAS) also in addition to cross sectional or case control study designs. Among participants of the Atherosclerosis Risk in Communities (ARIC) longitudinal cohort investigation (The ARIC Investigators, 1989) did not reveal a significant genome wide signals but suggested that 13 loci, including KCNK1, FBXO38, UHRF2, IL33, RUNX2, TRPS1, CAMTA1, and VAMP3, provide an evidence of association for red and orange complex microbiota except *Aggregatibacter actinomycetemcomitans* [2]. These results are further carried forward in another genome association study using MAGENTA (meta-analysis gene set enrichment of variant associations) approach to obtain gene-centric and gene set association results. The statistically significant association has been found for 6 genes; 4 with severe chronic periodontitis (*NIN, ABHD12B, WHAMM, AP3B2)* and 2 with high periodontal pathogen colonisation (red complex – *KCNK1*, *Porphyromonas gingivalis – DAB2IP).* The top gene sets included have been: for severe chronic periodontitis - endoplasmic reticulum membrane, cytochrome P450, microsome and oxidation reduction; for moderate chronic periodontitis - regulation of gene expression, zinc ion binding, BMP signalling pathway and ruffle; for periodontal pathogen colonisation-circadian clock system for red complex, G alpha Z signalling events for orange complex, KEGG mismatch repair for *Aggregatibacter actinomycetemcomitans* and protein binding for *Porphyromonas gingivalis* [25]. Thus, highlighted genes in previously identified loci and new candidate genes for explaining possible pathways associated with chronic periodontitis.

Recently genome wide association of chronic periodontitis is conducted by supplementing clinical data with biological intermediates of microbial burden and local inflammatory response with the formation of periodontal complex traits (PCTs). PCT1 has been characterised by a uniformly high pathogen load (Socransky trait), PCT4 with a mixed infection community whereas PCT3 and PCT5 have been dominated by *Aggregatibacter actinomycetemcomitans* and *Porphyromonas gingivalis*, respectively [10]. The genome-wide significant signals have been detected as mentioned in Table 1.

**Table 1:** Genome wide significant signals and the closest gene associated

Locus	SNP	Closest gene
PCT1 (Socransky Trait)		
16q11.2	rs1156327	CLEC19A (C-type lectin domain family 19 member A)
14q21	rs3811273	TRA (Transfer gene)
12q14	rs17184007	GGTA2P (Glycoprotein, Alpha- Galactosyltransferase 2 Pseudogene)
13q32.3	rs9557237	TM9SF2 (Transmembrane 9 Superfamily Member 2)
1q12	rs1633266	IFI16 (Interferon, Gamma-Inducible Protein 16)
3q12	rs17718700	RBMS3 (RNA Binding Motif Single Stranded Interacting Protein 3)
PCT3 (Aa Trait)		
4p15.33	rs4074082	C1QTNF7 (C1q and tumour necrosis factor-related protein 7)
8q24.3	rs9772881	TSNARE (T-SNARE Domain Containing)
PCT4 (Mixed Infection)		
7q21.1	rs10232172	HPVC1 (Human Papillomavirus (type 18) E5 Central Sequence Like 1)
PCT5(Pg Trait)		
12q14	rs7135417	SLC15A4 (Solute Carrier Family 15 Member 4)
11q14	rs6488099	PKP2 (plakophilin 2)
15q24	Rs904310	SNRPN (Small Nuclear Ribonucleoprotein Polypeptide N)

These highlighted loci mainly include genes associated with immune response and epithelial barrier function which enhance the disease susceptibility in the presence of a dysbiotic microbial structure [10]. However, these loci have not been associated with clinically defined disease which raises the possibility that these PCTs may be genetically tractable endophenotypes that nevertheless have little relevance to disease defined with clinical criteria alone. This has been suggested that the six PCTs, although having overlapping clinical presentations, may actually reflect six different conditions with distinct genetic risk profiles that may be discoverable only in the context of specific patterns of microbial dysbiosis and inflammatory response [10]. These new findings provide a logical sub-classification of disease based upon genetic and microbial-inflammatory signatures that warrants further validation.

Recently, a systematic review has been conducted on the periodontal infectogenomics included a total of 43 studies consisted of candidate genes and the above mentioned genome wide analyses and given a conclusion that there is no evidence yet that neither IL- 1 genetic polymorphisms nor any other investigated genetic polymorphisms are associated with presence and counts of subgingival microbiota. This is because of the heterogeneity and complexity of the study, case control study approach, small sample sizes and risk of bias analysis [80].

## Summary

To summarise, the host genotype may affect the colonisation pattern of subgingival species has been extensively discussed in the past few years. Nibali L et al. have reported that IL-6 hyperproducers (IL-6-174GG genotype subjects) show consistent association with *Aggregatibacter actinomycetemcomitans* detection in several independent studies in different populations. However, majority of the investigations of IL-1 and TNF-α polymorphisms have primarily reported association with subgingival colonisation of red and orange complex bacteria, but failed to give conclusive statements due to heterogeneity. The haplotypes of IL-4, IL-8 and IL-10 polymorphisms also has been associated with microbial colonisation. Other investigations about candidate genes vis IL-2, IL-12, IFN-γ, HLA class II, NF-κβ, vitamin D receptor, MMP-8, T-bet, apolipoprotein E and PPARγ polymorphisms had not documented any significant association with the pathogen colonisation. The findings from the published literature emphasises that in IL-2, IFN-γ, HLA class II and NF-κβ genotype needs further exploration of this association. Specifically, genotypes affecting pathogen detection receptors viz. CD14 260 CT + TT genotype has showed association with the red complex bacterias. MBL2 homozygote variant is found to be the only studied complement component suggesting the possibility to provoke the virulence of A. actinomycetemcomitans and Fcγ receptors reported the hyperactive phenotype of PMNs affecting the PMN function mainly phagocytosis and oxidative burst, resulting in severe microbial effect on periodontal tissue. Some of the polymorphisms associated with enhancement in special periodontal conditions as in case of gingival overgrowth subjects, the CD14 260 and IL-10 haplotype have been associated with the microbial colonisation of red complex bacteria. Since GWAS have been recently introduced in study of Periodontology, the evidence needs further exploration to define some conclusive association. So, the contemporary evidence available to explain the concept of periodontal imfectogenomics is complied as shown in Table 2.

**Table 2:** Summary of contemporary evidence related to periodontal infectogenomics

Authors	Study Design	Ethnicity	Number Of Patients	Clinical Groups	Analysed Gene Polymorphisms	Analysed Microbes	Associations
Complement System (MBL)							
Liukkonen A et al. 2017 [31]	CS	Finnish Study Population	222	Generalised Periodontitis, Localised Periodontitis, Periodontitis free	MBL2 (allele D, allele B, allele C) Grouped as: wild-type A/A, heterozygote A/O homozygote O/O	*Aa* *P. gingivalis*	MBL2 homozygote variant (O/O) type could provoke the virulence of *Aa*
TLR							
Kinane DF et al. 2006 [34]	In vitro	–	HGECs from healthy gingival tissues from 6 healthy subjects	Two HGECs from subjects heterozygous for the TLR4 polymorphism and four with the wild-type TLR4.	TLR4 Asp299Gly and Thr399Ile (Mutant type) TLR4 normal (Wild type)	*P. gingivalis*	Wild type TLR4 (Normal) appears more responsive to *P.gingivalis* than the mutant type
Holla LI et al. 2010 [35]	CC	Caucasian	481	CP and H	TLR2 2408G/A, i.e. Arg753Gln and -16934A/T TLR9-1486C/T, -1237C/T and 12848A/G	*Aa* *P. gingivalis* *P. intermedia* *T. forsythia* *T. denticola* *P. micros* *F. nucleatum*	Not significant
CD14							
Schulz S et al. 2008 [33]	CC	Caucasian	213	AgP, CP and H	CD14 -159C > T, TLR4 Asp299Gly, Thr399Ile	*P. gingivalis* *P. intermedia* *T. forsythia* *Aa* *T. denticola*	CD14 -159TT genotype + patients: < *P. intermedia* detection
Gong Y et al. 2013 [32]	CS	–	204	Renal transplant patients with and without cyclosporine A induced gingival overgrowth	CD14–260C > T	*P. gingivalis* *P. intermedia* *T. forsythia* *Aa* *T. denticola*	Gingival overgrowth patients with CD14–260 CT + TT: > detection of *P. gingivalis, T. forsythia*,*T. denticola* and red complex.
FcR							
Kobayashi T et al. 2000 [81]	CC	Japanese	33	CP and H	FcγRIIIb-NA1 and FcγRIIIb-NA2	*P.gingivalis*	CP patients with both FcγRIIIb-NA1/NA1 and FcγRIIIb-NA2/NA2 genotypes: lower stimulation index for IgG1- and IgG3-mediated phagocytosis in PMNs
Kaneko S et al. 2004 [41]	CC	Japanese and Caucasian	185	AgP	FcαRI nt324 A → G	*P. gingivalis*	FcαRI nt324 A/A in AgP: decreased phagocytosis of *P. gingivalis*
Wolf DL et al. 2006 [36]	CC	Caucasian	205	CP and H	FcγRIIIb NA1/NA2, FcγIIa 131R/H	19 bacterial stains	Not significant
Nicu EA et al. 2007 [37]	CS	Mixed	98	CP	FcγRIIa131H/R	*Aa*	In CP patients with FcγRIIa (H/H): increased phagocytosis, degraulation and elastase release after stimulation with *Aa*
Wang Y et al. 2012 [38]	CC	Japanese	119	CP and H (females post delivery)	FcγRIIbnt645 + 25A/G, FcγRIIb-nt646-184A/G,FcγRIIb-1232 T,FcγRIIa-R131H,FcγRIIIaV158F,FcγRIIIb-NA1/NA2	*P. gingivalis* *P. intermedia* *Aa*	Not significant
Sugita N et al. 2012 [39]	CS	Japanese	32	CP and H	FcγRIIb-nt645 + 25A/G	*P.gingivalis*	FcγRIIb-nt645 + 25AA genotype: < IgG4 levels produced against *P. gingivalis* sonicate and IgG2 produced against the *P. gingivalis* 40-kDa outer membrane protein (OMP)
IL-1							
Socransky SS et al. 2000 [42]	CS	–	108	CP	IL-1A *+* 4845 and IL-1B *+* 3954	40 taxa	IL-1genotype + subjects: > counts of *T. forsythia, T. denticola, F.nucleatum*, *F. periodonticum, Campylobacter gracilis, C. showae, Streptococcus constellatus. Streptococcus intermedius, Streptococcus gordonii* and 3 *Capnocytophage* species
Cullinan MP et al. 2001 [43]	L	Caucasian	295	CP	IL-1a + 4845 and IL-1B + 3954	*Aa* *P. gingivalis* *P. intermedia*	Not significant
Papapanou PN et al. 2001 [45]	CC	Caucasian	205	CP and H	IL-1A + 4845 and IL-1B + 3953	19 bacterial stains	Not significant
Jansson H et al. 2005 [77]	L	–	22	Patients with dental implants	IL-1α-889 and IL-1β + 3953	*P. gingivalis* *P. nigrescens* *Aa*	Not significant
Kowalski J et al. 2006 [48]	CS	–	16	CP	IL-1A-889 and IL-1B + 3953	*P. gingivalis* *P. intermedia* *T. forsythia* *Aa* *T. denticola* *F. nucleatum* *E. corrodens* *P. micros* *C. rectus*	IL-1 genotype + subjects: Higher total count of *C. Rectus*, red complex and orange complex bacteria IL-1genotype - subjects: Higher mean titre of *P. intermedia*
Agerbaek MR et al. 2006 [76]	CS	Caucasian	151	CP in supportive periodontal therapy	IL-1A + 4845 and IL-1B-3954	40 taxa	IL-1 genotype negative subjects: > total bacteria load and > levels of *Aa, E. nodatum, P. gingivalis, Streptococcus anginosus*
Kratka Z et al. 2007 [72]	L	–	20	AgP	IL-1A -889C/T and IL-1B + 3953C/T	*P. gingivalis* *P. intermedia* *T. forsythia* *Aa* *T. denticola*	Not significant
Ferreira SB et al. 2008 [47]	CC	Mixed	292	CP and H	IL-1β 3954	*P. gingivalis* *T. forsythia* *Aa* *T. denticola*	Not significant
Gonçalves L de S et al. 2009 [46]	CC	Mixed	105	CP and H (Grouped into HIV on HARRT and non HIV)	IL-1A + 4845 and IL-1B + 3954	33 bacterial species	Not significant
Schulz S et al. 2011 [71]	CC	Caucasian	248	AgP, CP and H	IL1α(rs180058),IL-1β (rs16944, rs1143634), IL-1R (rs2234650), and IL-1RA (rs315952)	*P. gingivalis* *P. intermedia* *T. forsythia* *Aa* *T. denticola*	Il-1α rs1800587, Il-1β rs 1,143,634 and composite genotype: > *Aa* detection in AgP group
Cantore S et al. 2014 [44]	CC	Italian Caucasian	195	H and CP	IL-1α + 4845 and IL-1β + 3954	Subgingival species	Not significant
Deppe H et al. 2015 [79]	Prospective	Caucasian	104	Type 2 diabetes mellitus patients and healthy controls	IL-1A, IL-1B and IL-1RN	Red, orange, green, yellow and purple complexes	Not significant
IL-2							
Reichert S et al. 2009 [49]	CC	Caucasian	200	AgP, CP and H	IL-2 -330 T/G and 166 G/T	*P. gingivalis* *P. intermedia* *T. forsythia* *Aa* *T. denticola*	IL-2-330, 166 TT-TT haplotype and 166TT: > detection of *P. gingivalis* and red complex IL-2 -330 TG: < *P. gingivalis* and red complex
IL-4							
Reichert S et al. 2011 [52]	CC	Caucasian	243	AgP, CP and H	IL-4RA Q551R	*P. gingivalis* *P. intermedia* *T. forsythia* *Aa* *T. denticola*	QR + RR polymorphism: Presence of *T. forsythia*
Finoti LS et al. 2013 [50]	CC	Caucasian	39	CP and H	IL-4 -590C/T, +33C/T and VNTR	*P. gingivalis* *T. forsythia* *T. denticola*	IL-4 TCI/CCI haplotype in CP: higher levels of *P. gingivalis, T. forsythia, T. denticola*
Bartova J et al. 2014 [51]	CC	–	62	CP and H	IL-4 -590c/T and intron 3 VNTR	*P. gingivalis* *P. intermedia* *T. forsythia* *Aa*	IL-4 -590CC and 11 of IL-4 VNTR: *T. forsythia* stimulates production of cytokines TNFα, IL-6, IL-10, IFNγ, IL-10, and IL-1*β* while *P. intermedia* affects the in vitro production of IL-6 and IL-10 CP.
IL-6							
Nibali L et al. 2007 [68]	CS	Mixed	45	AgP	IL-6 -174, Fcα, FcγRIIa, FcγRIIb, FcγRIIIa, FcγRIIIb, FPR, TNF and VDR	*Aa* *P. gingivalis* *T. forsythia*	IL-6- 174GG and Fcγ haplotypes: >*Aa* detection
Nibali L et al. 2008 [69]	CS	Mixed	107	AgP and CP	IL-1A -889, IL-1B -511, + 3954 IL-6 -174, − 572, − 1363, −1480, − 6106, TLR4–299,-399, TNFα − 308	*Aa* *P. gingivalis* *T. forsythia*	IL-6 -6106 AA and IL-6 haplotypes (−174G, -572C, − 1363G, -1480C, − 6106A): > detection of *Aa*
Nibali L et al. 2010 [57]	CS	Mixed	40	CP	IL-6 -174G > C	*Aa* *P. gingivalis*	IL-6- 174GG: >*Aa* detection
Nibali L et al. 2011 [70]	CS	Indian	251	H and with periodontal disease	IL-6 -174, − 572, − 1363, − 6106 and − 1480	40 taxa	IL-6- 174GG: > counts of *Aa* and detection and counts of *C. Sputigena*
Nibali L et al. 2013 [82]	L	Caucasian	12	AgP	IL-6 -1363, − 1480	*Aa*	IL6 haplotype: >counts of *Aa* before and after treatment
IL-8							
Linhartova PB et al. 2013 [55]	CC	Caucasian	492	AgP, CP and H	IL-8 -845C/T, −251A/T, + 396 G/T and + 781C/T	*P. gingivalis* *P. intermedia* *T. forsythia* *Aa* *T. denticola* *F. nucleatum* *P. micros*	IL8 − 251 T in AgP: > *A. actinomycetemcomitans detection* CC genotype of IL8 + 781 T/C variant in CP: < *T. forsythia* detection In non-periodontitis subjects with T allele of IL8 + 396G/T variant or TT genotype*: <F. nucleatum* detection.
Finoti LS et al. 2013 [54]	CS	Mixed	65	CP and H	IL-8 ATC/TTC	*P. gingivalis* *T. forsythia* *T. denticola*	Not significant
Finoti LS et al. 2013 [53]	CS	Mixed	30	CP and H	IL-8 ATC/TTC and AGT/TTC haplotype	*P. gingivalis* *T. forsythia* *T. denticola*	The diseased sites of AGT/TTC patients: harbour higher levels of *P. gingivalis, T. denticola, T. forsythia*, and red complex
IL-10							
Reichert S et al. 2008 [56]	CC	Caucasian	93	AgP, CP and H	IL-10 -1082G > A, -819C > T and -590C > A	*P. gingivalis* *P. intermedia* *T. forsythia* *Aa* *T. denticola*	IL-10 ACC, ATA and ACC/ATA haplotypes: < *P. intermedia* detection IL-10 GCC/GCC haplotype: > *P. intermedia* detection
Luo Y et al. 2013 [78]	CS	Chinese	202	Renal transplant patients with and without cyclosporine A induced gingival overgrowth	IL-10 -1082, −819 and − 592	*P. gingivalis* *P. intermedia* *T. forsythia* *Aa* *T. denticola*	Gingival overgrowth patients with ATA haplotype: higher detection and count of *P. gingivalis* and *T. denticola*
IFN-γ & IL-12							
Takeuchi-Hatanaka K et al., 2008 [73]	CS	Japanese	110	AgP, severe CP, mild CP and H	5′ flanking region of IL12RB2	*P. gingivalis* *P. intermedia* *T. forsythia* *Aa* *T. denticola* *F. nucleatum* *E. corrodens*	Higher serum IgG titres against periodontopathic bacteria in patients with variant alleles
Reichert S et al. 2008 [58]	CC	Caucasian	198	AgP, CP and H	IFN-γ 874 T/A IL-12 1188A/C	*P. gingivalis* *P. intermedia* *T. forsythia* *Aa* *T. denticola*	IFN-γ 874AA: < detection of *Aa* IFN-γ 874TA: > detection of *P. intermedia*
Holla LI et al. 2011 [59]	CC	Caucasian	498	CP and H	IFN-γ +874A/T	*P. gingivalis* *P. intermedia* *T. forsythia* *Aa* *T. denticola* *F. nucleatum* *P. micros*	Not significant
TNFα							
Schulz S et al. 2008 [60]	CC	Caucasian	175	AgP, CP and H	TNFα -308G > A and – 238G > A	*P. gingivalis* *P. intermedia* *T. forsythia* *Aa* *T. denticola*	TNFα308GG/238GG haplotype: > *P. intermedia* detection
Trombone APF et al. 2009 [62]	CC	Mixed	304	CP and H	TNFα -308G/A	*P. gingivalis* *T. forsythia* *Aa* *T. denticola*	Not significant
Schulz S et al. 2012 [61]	CS	Caucasian	942	Cp and H (All Coronary Artery Disease patients)	TNFα 308G > A and – 238G > A	*P. gingivalis* *P. intermedia* *T. forsythia* *Aa* *T. denticola* *P. micros* *F. nucleatum* *C. rectus* *E. nodatum* *E. corrodens* *C. sputigena*	TNFα-308 AG + AA genotype and A-allele: > *P. intermedia* detection
HLAII							
Shimomura-Kuroki J et al. 2009 [74]	CC	Japanese	64	AgP, CP and H	IL-1α −889, IL-1α + 4845, IL-1β + 3954 FcγRIIa-H/R131 HLA- DQB1	*P. gingivalis* *P. intermedia* *T. forsythia* *Aa* *T. denticola*	HLADQB1 BamHI sites in patients: > *T. forsythia* detection
NF-κβ							
Schulz S et al. 2010 [75]	CC	Caucasian	222	AgP, CP and H	TLR2 (Arg753Gln and Arg677Trp) NF-κβ -94ins/del ATTG	*P. gingivalis* *P. intermedia* *T. forsythia* *Aa* *T. denticola*	NF-κβ-94del/del: > *Aa* detection
VDR							
Borges et al. 2009 [64]	CC	Caucasian	60	CP and H	VDR TaqI	38 taxa	Not significant
T bet							
Cavalla et al. 2015 [63]	CC	Mixed	608	CP, CG and H	TBX21-1993 T/C	*P. gingivalis* *T. forsythia* *T. denticola*	Not significant
MMP8							
Holla LI et al. 2012 [65]	CC	Caucasian	619	CP and H	MMP8 (-799C/T and + 17C/G)	*P. gingivalis* *P. intermedia* *T. forsythia* *Aa* *T. denticola* *P. micros* *F. nucleatum*	Not significant
ApoE							
Linhartova PB et al. 2015 [66]	CC	Caucasian	469	CP and H	ApoE (rs429358C/T and rs7412C/T)	*P. gingivalis* *P. intermedia* *T. forsythia* *Aa* *T. denticola* *P. micros* *F. nucleatum*	Not significant
PPARγ							
Hirano E et al. 2010 [67]	CS	Japanese	130	CP and H All Pregnant Females Grouped as term birth and preterm birth	PPARγPro12Ala	*P. gingivalis* *P. intermedia* *T. forsythia* *Aa*	Not significant
GWAS							
Divaris K et al. 2012 [2]	–	Caucasian and Blacks	1020 white and 123 African American participants	Healthy to severe chronic periodontitis	2,178,777 SNPs	*C. rectus* *F. nucleatum* *P. nigrescens* *P. gingivalis* *T. forsythia* *T. denticola* *P. intermedia* *Aa*	Not a significant genome wide signals. But 13 loci, including KCNK1, FBXO38, UHRF2, IL33, RUNX2, TRPS1, CAMTA1, and VAMP3, provide an evidence of association for red and orange complex microbiota, but not for *Aa.*
Rhodin K et al. 2014 [25]	–	Caucasian	1020 + 4504 from two previously conducted GWAs	Healthy to severe chronic periodontitis	18,307 genes	*C. rectus* *F. nucleatum* *P. nigrescens* *P. gingivalis* *T. forsythia* *T. denticola* *P. intermedia* *Aa*	Statistically significant association for 6 genes – 4 with severe chronic periodontitis (*NIN, ABHD12B, WHAMM, AP3B2)* and 2 with high periodontal pathogen colonisation (red complex – *KCNK1*, *P. gingivalis – DAB2IP).*
Offenbacher S et al. 2016 [10]	–	–	975 European American For CP in the larger cohort (*n* = 821 severe CP, 2031 = moderate CP, 1914 = healthy/mild disease) and a German sample of 717 AgP cases and 4210 controls.	Healthy to severe chronic periodontitis and aggressive periodontitis	21,35,235 SNPs	8 periodontal pathogens divided into 6 PCTs with distinct microbial community as PCT1 with high pathogen load (Socransky trait), PCT4 with a mixed infection, PCT3, PCT5 dominated by *Aa* and *P. gingivalis*, respectively.	Genome-wide significant signals for PCT1 (CLEC19A, TRA, GGTA2P, TM9SF2, IFI16, RBMS3), PCT4 (HPVC1) and PCT5 (SLC15A4, PKP2, SNRPN). Overall, the highlighted loci included genes associated with immune response and epithelial barrier function.
Systematic review							
Nibali L et al. 2016 [80]	–	–	43 studies of candidate genes and two GWAS	Healthy to severe chronic periodontitis and aggressive periodontitis	–	Periodontal Pathogens	No evidence yet that neither IL-1 genetic polymorphisms nor any other investigated genetic polymorphisms are associated with presence and counts of subgingival microbiota.

*Aa* *Aggregatibacter actinomycetemcomitans*, *AgP* Aggressive Periodontitis; CS: Cross Sectional, *CC* Case Control, *H* Healthy, *CG* Chronic Gingivitis, *HGECs* Human Primary Gingival Epithelial Cultures, *CP* Chronic Periodontitis, *L* Longitudinal

The major issues concerned to the study of infectogenomics is the difficulty in comprehensively examining the subgingival microbiota which is further complicated by nature of microbial infection, biofilm type formed where both the symbiotic and exogenous bacteria are organised which behave as part of a complex and polymicrobial nature of periodontal infection and due to inadequate knowledge of specific host genetic factors that are likely to affect the subgingival microbiota.

## Conclusion

The functional genomics of host has crucial importance while analysing host- pathogen interactions in the pathogenesis of periodontal disease. An increased understanding of the genetics underpinning of interactions between the host and exogenous or symbiotic bacterial communities has the potential to advance our knowledge not only of periodontitis, but also of other chronic inflammatory and microbiome-related diseases. Several risk loci identified may offer promising leads for further exploration and mechanistic studies which have the potential to unveil pathways and mechanisms that direct the host’s symbiosis with healthy microflora to dysbiosis state which may predispose to the disease state. Therefore, infectogenomics may serve as a useful model to study the relationship between host genome and microbial challenge. Further exploration of the concept is essential to identify infectious states, to understand the host response, to predict disease outcomes, to monitor responses to antimicrobial therapies and to indicate promising new types of treatment.

## Future directions

The field of periodontal infectogenomics can determine different pathogenic pathways in different forms of periodontitis, and possibly assist in early prevention and management of disease. Additional multicentre studies based on large population samples in different populations and with high-quality phenotypes need to be conducted worldwide to identify the human genetic factors that predispose to invasion by pathogens and to their proliferation. Changes in gene expression profiles can also determine the type of pathogen present. Thus, gene expression patterns in the blood could serve as a window into the pathogenesis and diagnosis of infectious diseases. Advances in gene expression profiling may possibly provide the chance for adjunctive pharmacological treatment. Further research is needed to validate the biologic basis for genetic susceptibility testing, to evaluate the ability of different genotypes to predict disease initiation and to evaluate the effectiveness of genotyping in making diagnostic or treatment intervention strategies, especially in dental new age tissue engineering approach. However, the consideration of specific microbiota with distinct exposure is consistent with the paradigm of periodontal medicine which may provide an insight into the new alternative connection of oral-systemic diseases.

## Abbreviations

Aa: Aggregatibacter actinomycetemcomitans
ABHD12B: Abhydrolase Domain Containing 12B
AgP: Aggressive Periodontitis
AP3B2: Adapter Related Protein Complex 3 Beta 2 Subunit
BMP: Bone Morphogenetic Protein
CC: Case Control
CCR5: Chemokine Receptor 5
CG: Chronic Gingivitis
CP: Chronic Periodontitis
CS: Cross Sectional
DAB2IP: Disabled Homolog 2 Interacting Protein
H: Healthy
HGECs: Human Primary Gingival Epithelial Cultures
HIV: Human Immunodeficiency Virus
HLA: Human Leukocyte Antigen
IFN-γ: Interferon Gamma
IL-4RA: Interleukin -4 Receptor α Chain 43
KCNK1: Potassium Two Pore Domain Channel Subfamily K Member 1
L: Longitudinal.
MBL: Mannose Binding Ligand
MHC II: Major Histocompatibility Complex II
MMP 8: Matrix Metalloproteinases 8
NF-κβ: Nuclear Factor Kappa β
PAMPs: Pathogen Associated Molecular Patterns
PRPs: Peptidoglycan Recognition Proteins
RUNX2: Runt Related Transcription Factor 2
SNPs: Single Nucleotide Polymorphisms
Th: T Helper Cells
TNF-α: Tumor Necrosis Factor Alpha
TRPS1: Transcription Repressor GATA Binding 1
UHRF2: Ubiquitin like PHD and Ring Finger Domain 2
VAMP3: Vesicle associated Membrane Protein 3
WHAMM: WAS Protein Homolog Associated with Actin, Golgi Membranes and Microtubules
